# Biomedical Research Programs at Present and Future High-Energy Particle Accelerators

**DOI:** 10.3389/fphy.2020.00380

**Published:** 2020-10-16

**Authors:** Vincenzo Patera, Yolanda Prezado, Faical Azaiez, Giuseppe Battistoni, Diego Bettoni, Sytze Brandenburg, Aleksandr Bugay, Giacomo Cuttone, Denis Dauvergne, Gilles de France, Christian Graeff, Thomas Haberer, Taku Inaniwa, Sebastien Incerti, Elena Nasonova, Alahari Navin, Marco Pullia, Sandro Rossi, Charlot Vandevoorde, Marco Durante

**Affiliations:** 1 Dipartimento di Scienze di Base e Applicate per l’Ingegneria, University “La Sapienza”, Rome, Italy; 2 Institut Curie, University Paris Saclay, Orsay, France; 3 iThemba LABS, NRF, Cape Town, South Africa; 4 TIFPA, INFN, Trento, Italy; 5 LNL, INFN, Legnaro, Italy; 6 KVI-CART, University of Groningen, Groningen, Netherlands; 7 JINR, Dubna, Russia; 8 LNS, INFN, Catania, Italy; 9 Université Grenoble-Alpes, CNRS/IN2P3, UMR5821, LPSC, GDR MI2B, LabEx PRIMES, Grenoble, France; 10 GANIL, Caen, France; 11 Biophysics Department, GSI Helmholtzzentrum für Schwerionenforschung, Darmstadt, Germany; 12 HIT, University of Heidelberg, Heidelberg, Germany; 13 NIRS, QST, Chiba, Japan; 14 Université de Bordeaux, CNRS/IN2P3, UMR5797, Centre d’Études Nucléaires de Bordeaux Gradignan, Gradignan, France; 15 CNAO, Pavia, Italy; 16 Institut für Festkörperphysik, Technische Universität Darmstadt, Darmstadt, Germany

**Keywords:** accelerators, particle therapy, space radiation protection, high-energy ions, biomedical research

## Abstract

Biomedical applications at high-energy particle accelerators have always been an important section of the applied nuclear physics research. Several new facilities are now under constructions or undergoing major upgrades. While the main goal of these facilities is often basic research in nuclear physics, they acknowledge the importance of including biomedical research programs and of interacting with other medical accelerator facilities providing patient treatments. To harmonize the programs, avoid duplications, and foster collaboration and synergism, the International Biophysics Collaboration is providing a platform to several accelerator centers with interest in biomedical research. In this paper, we summarize the programs of various facilities in the running, upgrade, or construction phase.

## Introduction

Particle accelerators have provided an extensive contribution to research beyond particle and nuclear physics. Astrophysics, atomic physics, plasma physics, materials research, environmental science, archaeometry, homeland security, space radiation research, biology, and medicine largely use and benefit from particle accelerators [1]. Biomedical applications are particularly important, for their impact on societal health [2]. One of the main medical applications of accelerators is certainly the production of radioisotopes to be used for imaging, therapy, or both (theranostics) [3–5]. Accelerators also spawned charged-particle therapy, a technique for cancer treatments that exploits the Bragg peak of charged particles and can reduce toxicity and improve local control compared to conventional X-ray radiotherapy [6]. Fast neutrons have been used in the past for cancer therapy but then dismissed because of inacceptable toxicities [7]. Epithermal and thermal neutrons can, however, effectively kill tumors loaded with ^10^B, the so-called boron-neutron capture therapy (BNCT) [8, 9]. BNCT has been hampered by the necessity of using nuclear reactors for treatment but is now revived by the perspective of using dedicated proton accelerators [10]. Cyclotrons and synchrotrons for charged-particle therapy are blooming worldwide [11–13], and many of these centers have intense preclinical research programs [14]. Research in space radiation protection also needs accelerators to simulate the cosmic radiation that astronauts find in the space environment [15–19]. In fact, most of our knowledge on radiation risk in space comes from experiments at particle accelerators [20, 21].

**Figure 1:**
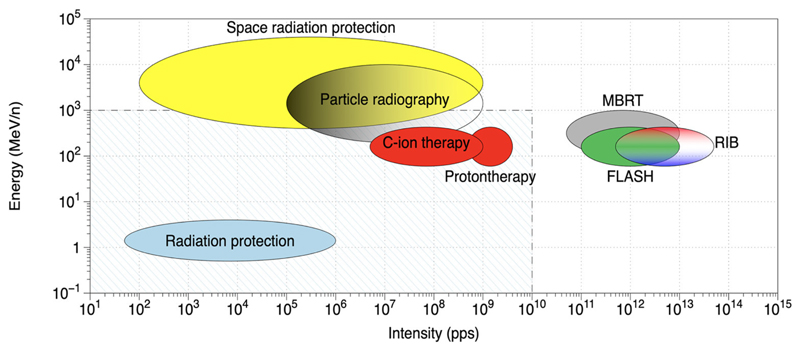
Radiation research at accelerators. The shaded region includes values of energy and intensities covered by the present accelerators. MBRT, minibeam radiotherapy; RIB, radioactive ion beams; FLASH, high-dose-rate radiotherapy.

Many new large-scale accelerators are under construction worldwide, with the primary goal of basic research in nuclear physics, generally exploring the region far from stability [22]. Most of the accelerators centers have ambitious biomedical research programs that are innovative and potentially can lead to breakthrough discoveries thanks to the characteristics of the new facilities, generally with higher intensity and energy than current accelerators have [23]. Figure 1 shows some of the opportunities that can exploit the characteristics of new accelerators or the upgrade of existing facilities. High energy is obviously important for space radiation research, because cosmic rays have energies up to TeV [24, 25] but can also be useful for particle radiography [26], an important technique to reduce range uncertainty in particle therapy. High intensity can potentially be a major breakthrough in particle therapy: ultrafast treatments are convenient for patient welfare and for clinical workflow and can mitigate the problem of moving targets [27]. Recent results with electron beams suggest that dose rates exceeding 40 Gy/s reduce toxicity in the normal tissue while maintaining tumor local control (FLASH radiotherapy) [28, 29]. High intensity is also useful for spatially fractionated radiotherapy using protons [30] or heavier ions [31], a method that largely reduces normal tissue toxicity in animal models [32–34]. Finally, radioactive ion beams (RIB), one of the main nuclear physics topics that justify the construction of new nuclear physics facilities [35], are potentially an extraordinary tool for therapy as they allow the online visualization of beams during irradiation [36].

While all these research programs are exciting, it is important to avoid duplications, exploit synergism, and foster collaborations and strong links between clinical accelerators and nuclear physics accelerators planning applied biomedical research. For these reasons, many facilities have joined the International Biophysics Collaboration [37] that had a first meeting in Darmstadt in May 2019 [38]. Here, we present the biomedical research programs of several accelerator facilities that have joined the Biophysics Collaboration.

## Biomedical Research Programs at Particle Accelerators

### Fair

**Figure 2:**
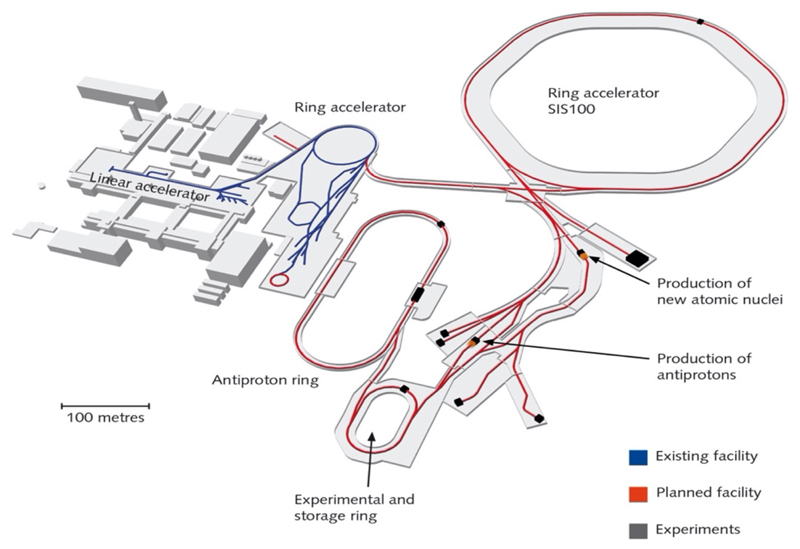
Layout of the FAIR facility under construction in Darmstadt (Germany).

The Facility for Antiprotons and Ion Research (FAIR) is currently under construction in Darmstadt [39]. As shown in Figure 2, the current SIS18 synchrotron (18 Tm) at GSI will become the injector of the new SIS100 (100 Tm) ring. All ions from H to U can be accelerated up to around 10 GeV/n. FAIR will also reach intensities up to ×10,000 higher than those currently available at GSI, and this intensity upgrade is already ongoing at SIS18 in the framework of the so-called FAIR-phase-0 [40]. While the official opening of the SIS100 is slated for 2017, research is currently ongoing within the FAIR-phase-0. Research activity at FAIR is structured into four pillars: NuSTAR, CBM, PANDA, and APPA. APPA deals with applied research (biophysics and materials research) and atomic and plasma physics [41]. FAIR is a user facility, and research is proposed by collaborations. The Biophysics Collaboration is indeed based at FAIR^1^ but, unlike other collaborations, includes other accelerator facilities, and aims at a distributed research program.

**Figure 3:**
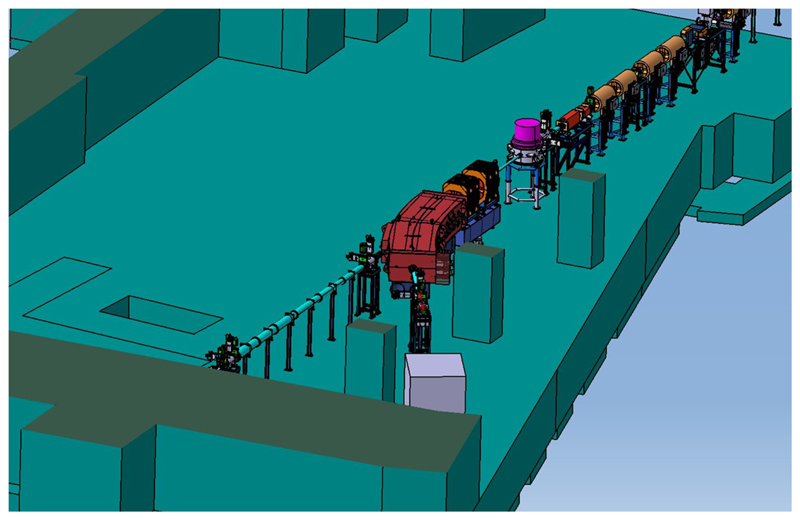
Beamline for BIOMAT applications in the APPA cave at FAIR.

The biophysics research program at FAIR imposes on the exceptional experience of the Biophysics Department in both heavy-ion therapy and space radiation research [Kraft et al. submitted]. In fact, GSI was the first center in Europe to treat patients with accelerated ^12^C ions [42] and is currently the reference center of ESA for the ground-based research program [16] called IBER^2^. With the end of the therapy in 2007, GSI activity focused on heavy-ion basic research, with applications to therapy and space radiation protection. Research at FAIR will therefore continue in these directions, according to the new opportunities that the SIS100 energies and the upgraded intensities offer (Figure 1). The new research programs include the construction of a galactic cosmic ray simulator [43], high-energy particle radiography [44], FLASH irradiations with heavy ions [45], and testing of carbon and oxygen radioactive isotopes for therapy and simultaneous imaging by PET [36], a program that has been supported by a recent ERC Advanced Grant (BARB)^3^. The Biophysics Department will benefit from FAIR with a new experimental vault, the APPA cave (Figure 3), where especially high-energy space radiation protection experiments will be performed.

## NICA

**Figure 4:**
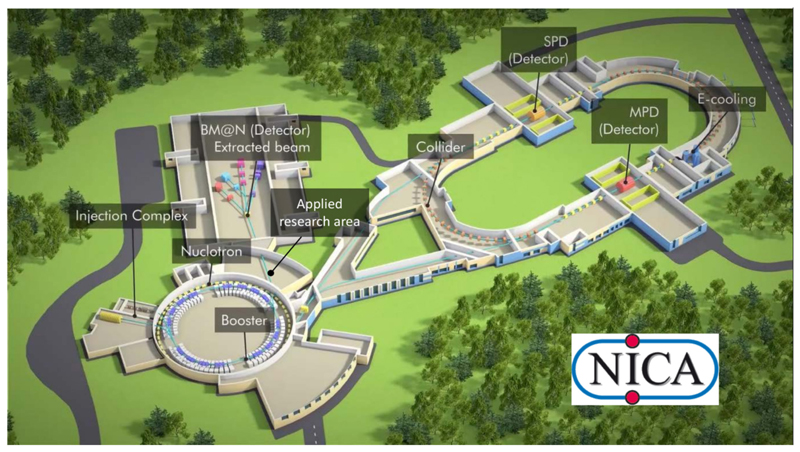
Layout of the NICA facility under construction in Dubna (Russia).

The Nuclotron-based Ion Collider fAcility (NICA) is a new accelerator facility designed at the Joint Institute for Nuclear Research (JINR, Dubna, Russia) to study properties of dense baryonic matter [46, 47]. The NICA facility (Figure 4) includes the injection complex, a new superconducting booster synchrotron, the existing modernized superconducting heavy-ion synchrotron “Nuclotron,” a new collider with two superconducting storage rings and with two interaction points [one for heavy-ion studies with the multipurpose detector (MPD) and another for polarized beams for the spin physics detector (SPD) experiment], an electron cooling system, new beam transfer channels, and the experimental zone for extracted beams with a Baryonic Matter at Nuclotron (BM@N) detector. The main goal of the project is the study of hot and dense strongly interacting matter in heavy-ion (up to Au) collisions. A study of spin physics with extracted and colliding beams of polarized deuterons and protons is also planned. Gold ions will be accelerated up to a kinetic energy of 4.5 GeV/u; the polarized protons, up to 12.6 GeV. Two modes of operation are foreseen: collider mode and extracted beams. The proposed program allows one to search for possible signs of phase transitions and critical phenomena as well as to shed light on the problem of the nucleon spin structure. For applied physics research, three new experimental areas are planned. Topics of interest are radiobiology and particle therapy, cosmic ray simulation, radiation hardness of electronic devices, novel technologies in materials science, and nuclear energetics. Ion beams with an energy of 250–800 MeV/u extracted from Nuclotron will be used for these experiments. The commissioning of beamlines and experimental stations for applied research as a part of basic NICA configuration is expected in 2022.

The biomedical research program carried out by the Laboratory of Radiation Biology (LRB) at the NICA complex will be focused on studying heavy-ion action at the molecular, cellular, tissue, and organism levels of biological organization. Primary attention will be paid to research on experimental animals’ central nervous system (CNS) disorders because the CNS must be considered a critical system when evaluating the radiation exposure risk for the interplanetary mission crews and considering the possible side effects of the radiotherapy of brain tumors. The main advantage of LRB and NICA is an excellent opportunity to perform large-scale *in vivo* animal exposures in collaboration with leading Russian experts in this field, who have all the necessary licenses. The research on rodents includes behavioral studies, pathomorphological studies of irradiated brain structures with the aim of modern immunohistochemical methods and morphometry, cytogenetics, and neurochemical and electrophysiological studies. Worldwide unique experiments on primates for the estimation of radiation risks of CNS disorders and carcinogenesis are in progress at the LRB. The LRB also develops a hierarchy of mathematical models to simulate radiation-induced pathologies at different organization levels and time scales. In addition to the traditional Monte Carlo technique, the LRB’s approach involves computational methods from different knowledge areas (molecular dynamics and simulation of brain neural networks). The radiation research program at NICA can contribute to a better reproduction of the space environment. The LRB has proposed a novel Nuclotronbased technique of modeling radiation fields with continuous particle energy spectra generated by galactic cosmic rays inside spacecraft in deep space.

A huge amount of experimental work has to be done at accelerators worldwide to understand how heavy charged particles may disturb the CNS performance after cancer therapy or during space flights. Certainly, there is a strong need for broad international collaboration in this field.

### iThemba Labs

With more than 30 years of operation of the separated sector cyclotron, the iThemba Laboratory for Accelerator Based Sciences (LABS) is the largest facility for accelerator-based sciences in the southern hemisphere. It is one of the research infrastructure platforms of the National Research Foundation (NRF) in South Africa, with the main goals of supporting research of strategic importance, training the future research workforce, and providing access to unique infrastructure for national and international users.

The facility has a long history and expertise in radiation biophysics research, which went hand in hand with the start of the particle therapy program in 1988 with a 66 MeV p + Be isocentric neutron therapy system and a fixed 200 MeV proton therapy facility [48]. In the first decades, the research program was dominated by clinical research and the development and optimization of particle therapy treatment modalities. Today, the new Radiation Biophysics Division is driving a multidisciplinary research program that converges the existing expertise in the field of radiation biology and medical physics, to investigate the relationship between radiation quality and biological effects. Researchers can make use of the well-characterized 200 MeV proton beamline, as well as the neutron beamlines available at iThemba LABS. The latter includes a rather unique quasi mono-energetic neutron metrology beamline, with beam energies ranging from 30 to 200 MeV, using (p, n) reactions on thin Li and Be targets [49]. Currently, very little information is available on the biological effects of high-energy neutrons (>20 MeV) that are most pertinent to applications in civil aviation, future manned space missions, and particle therapy. Therefore, the well-characterized neutron fields at iThemba LABS will be of growing importance in the coming years, to fill this gap in an attempt to decrease the existing uncertainties on neutron weighting factors and the relative biological effectiveness at higher neutron energies.

**Figure 5:**
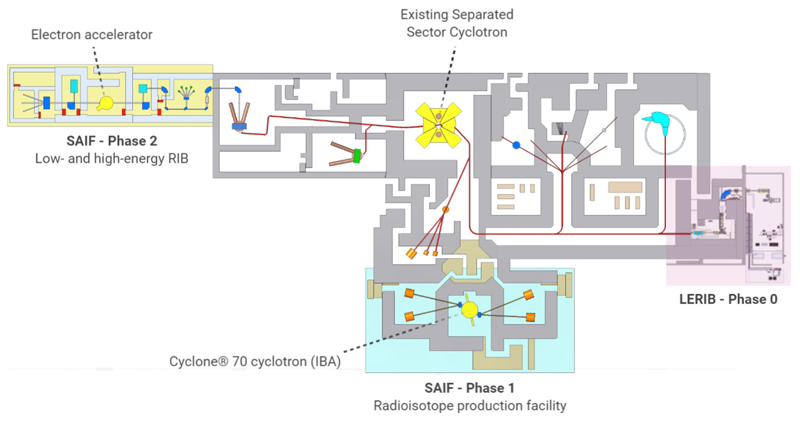
Layout of the main facility at iThemba LABS and its future developments. SAIF phase 1 includes phase 0 of the LERIB facility (shaded in pink) and the new 70 MeV cyclotron with its target stations (shaded in blue). The second phase of the project includes the installation of a Rhodotron in the area shaded in yellow for the production of radioactive ion beams (RIB).

Next to research projects with external particle beams, there is a growing interest in radioisotope research. This is attributable to the launch of the South African Isotope Facility (SAIF) at iThemba LABS in 2019, which includes the acquisition of IBA’s Cyclone^Ⓡ^ 70 cyclotron (Figure 5) [50, 51]. The advent of the new 70 MeV cyclotron at iThemba LABS will not only increase South Africa’s radioisotope production capacity but will also boost research into new solutions for nuclear medicine applications. This will be achieved through the optimization of isotope production processes, research in radiochemistry, radiolabeling, and preclinical radiobiological studies on newly developed radiopharmaceuticals. In the coming years, a strong focus will go to the development of new theranostic radiopharmaceuticals and the production of astatine-211, a promising isotope for targeted α-particle therapy [52].

On the one hand, the research program of the Radiation Biophysics Division at iThemba LABS can be summarized in cancer detection and therapy projects, with a main focus on radioisotopes and particle therapy. This comprises studies on systemic effects and the tumor microenvironment, such hypoxia and tumor angiogenesis. On the other hand, there is a set of research projects linked to radiation protection, which includes biological dosimetry projects and the implementation and validation of the first ground-based setup for space radiobiology research in Africa. For all projects, microdosimetry and Monte Carlo simulations remain vital tools, in order to assess the microscopic patterns of energy deposition by radiation, which ultimately govern the observed biological effects [53]. Lastly, the location of iThemba LABS provides the advantage to conduct projects that are unique to Africa, including studies on potential inter-ethnic differences in radiation sensitivity and the cancer resistance of large long-living mammals, such as African elephants [54].

The SAIF project at iThemba LABS, as outlined in Figure 5, is designed in two phases. Phase 1 consists of a radioisotope facility with four production targets and the initial phase (phase 0) of the Low Energy Radioactive Ion Beam (LERIB) facility. Here, the high-intensity proton beam from the SSC (up to 250 μA) will be used as a driver for the Isotope Separation On-Line (ISOL) production of radioactive isotopes of special interest in, for example, the study of neutron-rich nuclei involved in the rprocess. Phase 2 will comprise the building of a new driver for the production of RIB, based on a high-intensity electron beam and the photo-fission method for the production of neutron-rich exotic isotopes which will be used as higher-intensity low-energy RIB but also accelerated high-intensity RIB using the SSC as a post-accelerator.

## HIMAC

In the National Institute of Radiological Sciences (NIRS), carbon ion radiotherapy has been conducted since 1994 using the Heavy-Ion Medical Accelerator in Chiba (HIMAC). During the past 25 years, this radiotherapy has been applied to various tumors, and the optimum dose-fractionation protocols have been developed for these tumors through dose-escalation clinical trials [55–58]. To date, more than 12,000 patients have been treated with the HIMAC. Besides the clinical studies, various physical studies have been conducted to develop new treatment methods and devices such as respiratory gating [59], layer stacking [60], 3D pencil beam scanning [61], and a superconducting rotating gantry [62]. For further development of charged-particle therapy, the NIRS initiated a new research project referred to as “Quantum Scalpel.” The Quantum Scalpel consists mainly of two research topics. The first topic is downsizing and cost reduction of the treatment facility. By combining high-power laser and superconducting magnet technologies, the facility size will be reduced to ~1/6 that of the HIMAC, i.e., 20 × 10 m^2^. The second topic is maximizing the clinical effects and minimizing the treatment period. For this, researchers in the Department of Accelerator and Medical Physics are developing a hypo-fractionated multi-ion radiotherapy (HFMIT) in which several ion species are delivered in one treatment session to optimize the dose and linear energy transfer (LET) distributions simultaneously [63]. Clinical trials of the HFMIT will start in 2022 following a series of commissioning tests. In other projects, emerging technologies such as immunotherapy, magneto-particle therapy [64], and FLASH radiotherapy [29] have been found to show enhanced novel effects with charged-particle beams. Investigations at the NIRS continue on all of these technologies.

## GANIL and MI2B

**Figure 6:**
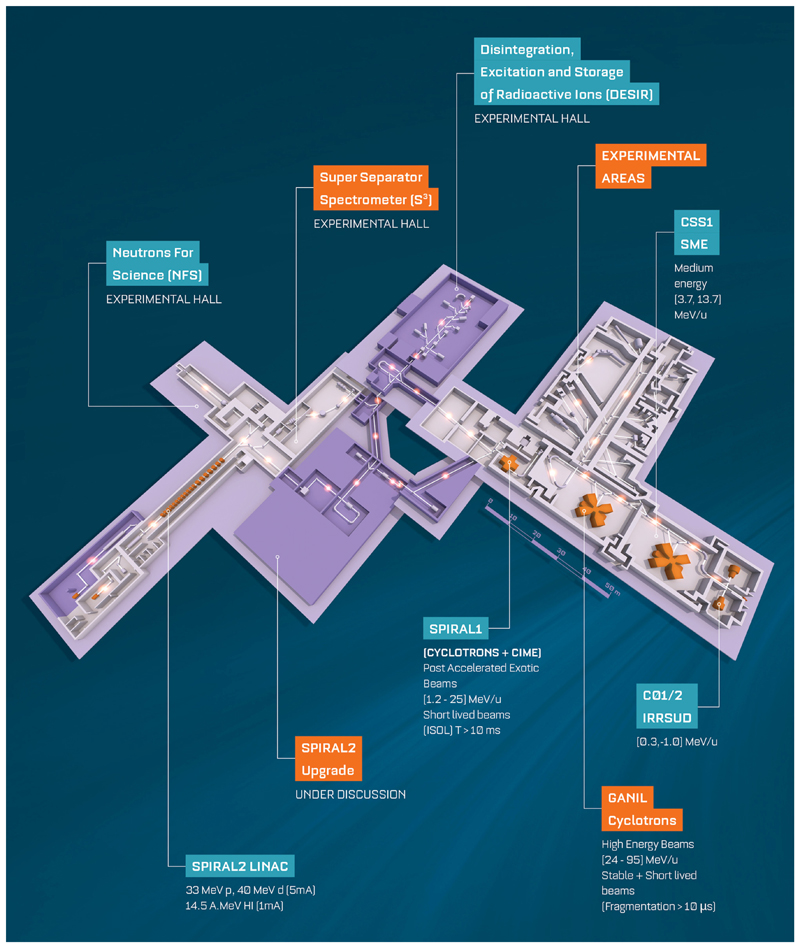
Layout of the GANIL-SPIRAL2 facility in Caen (France).

The largest facility for nuclear physics in France located in Caen is jointly run by CEA and CNRS. GANIL as well as its major upgrade SPIRAL2 (Figure 6) is engaged in research with ion beams with the main focus of the laboratory being fundamental nuclear physics. This is supplemented by strong programs in accelerator-based atomic physics, condensed matter, radiobiology, and industrial applications. The intensity and variety of beams delivered by the cyclotrons and the superconducting linear accelerator and the associated state-of-the-art scientific instruments make GANIL-SPIRAL2 a unique and outstanding multidisciplinary facility [65]. GANIL-SPIRAL2 is the only facility in the world today which provides high-intensity stable beams, beams of short-lived nuclei (RIB) produced both by the ISOL technique and by the in-flight separation technique and intermediate energy neutron beams [66]. The large heavy-ion accelerator complex of five cyclotrons delivers stable beams (carbon to uranium) from energies around 1–95 MeV per unit mass with current up to 10 μA. Fragmentation beams range from light to medium mass nuclei. The reaccelerated beams produced using SPIRAL1 beams range from 1.2 to 25 MeV/A for around 35 isotopes today. A new ion source has been commissioned, so more new RIB for different elements are available, and more will be available^4^ in the near future. The new superconducting LINAC will provide the most intense beams from protons to Ni up to 14.5 MeV per unit mass. Continuous and quasi-mono-energetic beams of neutrons will be available. The flux at NFS will be up to 2 orders of magnitude higher than those of other existing time-of-flight facilities for a part spectrum in the 1–40 MeV range. The latter will open new and unique avenues.

Materials science research at GANIL is studied using a large range of energies and beams along with versatile tools. High-energy ions provide quality beams for studies on nanostructuration of selective membranes and sensor developments based on topical 2D materials (graphene, MoS2, etc.). Exploiting time/depth-resolved characterizations to their limit, the sensitivity of functional inorganic materials to dense electronic excitations is studied. Advanced experimental setups also provide *in situ* analysis for organic polymers (CESIR or CASIMIR) or astro-ices (IGLIAS) as simulators for alpha radiation, cosmic rays, or solar winds.

The relevant biomedical activities span a variety of topics. Measurements of double differential cross section for charged particles with 95 MeV/A C beams on targets of various elements that are relevant to hadron therapy were performed. Irradiation and hardening of electronic components for space are performed using heavy and energetic ions. The studies include single-event effect (SEE) to improve the architectures and define testing standards used in space. Dedicated equipment for irradiation of polymeric films allow industrial production with various ion track densities and ultimately very fine and uniform filters. The LARIA center at GANIL studies various aspects related to the study and understanding of the biological effects related to direct and indirect (bystander) impacts by carbon beams in cancer treatment. The topics range from understanding differential cellular responses of radioresistant tumors to conventional radiotherapy and hadrontherapy to exploring the fundamental mechanisms of communication between irradiated and normal cells, etc. The facilities for these activities include cell culture room, two sterile hoods, four CO_2_ incubators, a microscope, water baths, centrifuges, etc. All the above activities are run at the cyclotrons. Light-ion beams from the LINAC, like alpha and ^6,7^Li beam on Pb and Bi targets, will be used to perform R&D on the production of innovative radioelements for nuclear medicine and in particular alpha emitters. This will consist in cross-section measurements to determine the optimum energies maximizing the cross section for the nuclei of interest (e.g., the promising ^211^At) [67] while minimizing those for nearby contaminants (^210^At and ^209,210^Po in the case of ^211^At); to develop high-power target stations to sustain the very high beam current from the new LINAC; and to find new and promising production routes. The NFS facility can be used for irradiation of cells, for characterization of detectors, and also for the study of the single-event defects.

**Figure 7:**
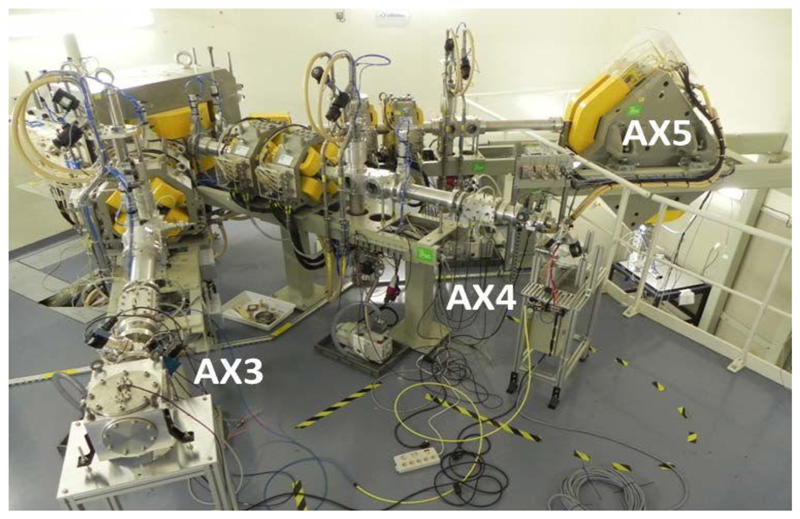
Experimental cave at ARRONAX (Nantes, France). AX5 is a vertical irradiation beamline.

In France, several irradiation facilities for biomedical applications are being coordinated by CNRS within the so-called “*Groupement de Recherche* MI2B.”^5^ MI2B animates a national network of clinical-based and academic research-based irradiation facilities called ResPlanDir, dedicated to dosimetry, instrumentation, and radiobiology, by supporting harmonization of practices. Among the various irradiation modalities, one should mention the availability of a complete panel of proton and light-ion irradiation platforms: AIFIRA at CENBG-Bordeaux^6^ proposes up to 3.5 MeV proton or alpha particle microbeams (size of typically 1.5 μm FWHM in air) equipped with an online microscope. This makes possible the selective irradiation of single-layered cells. CYRCé at IPHC-Strasbourg^7^ is a combined platform for radioisotope production working for academic research, with a newly functional proton irradiation platform with energy ranging from keV to 24 MeV^8^ for cell or small-animal irradiation (possibility to tune a spread-out Bragg peak up to 6 mm), a biological laboratory with small animals, and preclinical imaging (PET and SPECT). The ARRONAX facility^9^ is a combined research and innovative radioisotope production facility, delivering protons (35 and 70 MeV), deuterons (15 and 35 MeV), and alpha particles (70 MeV). A dedicated experimental irradiation room (Figure 7) has been equipped for physics and materials science experiments and cell irradiation by means of a vertical beamline.

## INFN and CNAO

The National Institute for Nuclear Physics (INFN) in Italy has several accelerator facilities with biomedical applications, including Laboratori Nazionali del Sud (LNS) in Catania [68], the first center in Italy to treat patients with proton therapy for eye tumors, and the Trento Institute for Fundamental Physics and Applications (TIFPA), where an experimental vault [69] with two beamlines delivering protons with energies up to 230 MeV is available in the local proton therapy center, where two other rooms are equipped with isocentric gantries for treating patients.

The INFN National Laboratories of Legnaro (LNL) are devoted to the study of fundamental nuclear physics and astrophysics together with the development of technologies relevant to these disciplines. Ever since its foundation, LNL has carried out a significant applied physics research, developing very relevant programs in the biomedical field with the existing LNL accelerators as well as the future SPES facility. Applications of ion beams in multidisciplinary physics are a long-standing tradition of LNL. These activities are carried out mainly at the AN2000^10^ and CN^11^ Van de Graaff accelerators and partly at the Tandem^12^. The CN (1–6 MV) and AN2000 (0.2–2.2 MV) provide a total of 12 beamlines and deliver around 2,700 h/year of beamtime (^1^H, ^2^H, ^3^He, ^4^He, ^14^N, and ^15^N). The main activities at the AN and the CN in the field of interdisciplinary physics are radiobiology [70, 71], dosimetry, materials microanalysis with IBA methods, study of novel neutron detectors based on innovative materials, single-ion irradiation for quantum technologies, and HpGe, Si, and diamond detector characterization.

LNL has a very long and strong tradition in the field of microdosimetry and nanodosimetry [72, Colautti et al. submitted]. In particular, Legnaro is one of the leading laboratories for the construction of miniaturized tissue equivalent proportional counters. Legnaro is in contact with various radiotherapy centers for the supply of these detectors or the microdosimetric characterization of therapeutic beams (Detector/MedAustron, SCK, JINR). These detectors can be used for quality assurance of treatment planning systems which include linear energy transfer calculations. In the field of nanodosimetry, STARTRACK is one of three detectors in the world for measuring the stochastics of radiation interaction at the DNA level [73]. It is installed and running at Tandem. A portable version is under construction.

**Figure 8:**
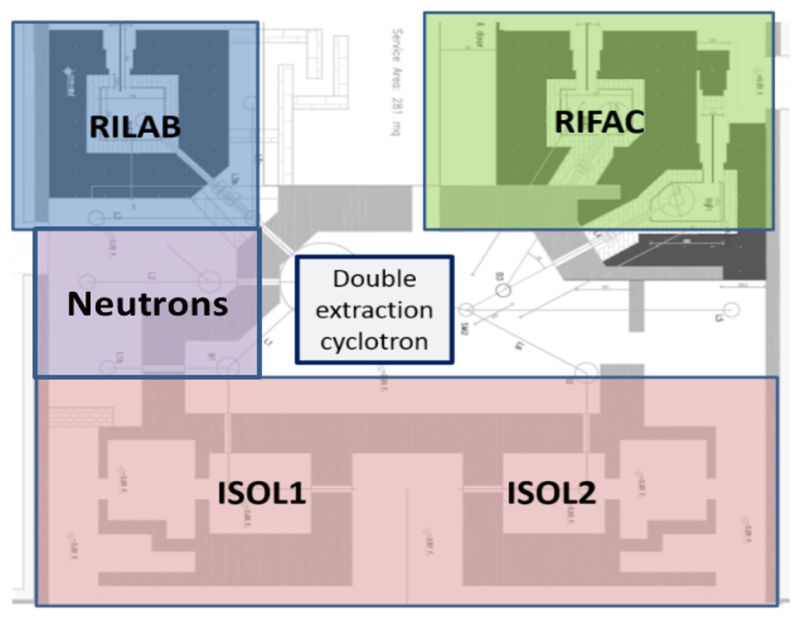
Layout of the SPES facility under construction in Legnaro (Italy).

Selective Production of Exotic Species (SPES)^13^ is a second-generation ISOL facility on which the short- and long-term strategies of the laboratory are centered [74]. It is an interdisciplinary project, ranging over nuclear physics, nuclear medicine, and materials science. SPES will provide a RIB facility for the study of neutron-rich unstable nuclei of interest to nuclear and astronuclear physics research [75]. At the same time, it will host a laboratory for research and production of radioisotopes to be applied in nuclear medicine. SPES is based on a dual-exit high-current cyclotron, with proton beam energy ranging between 35 and 70 MeV and a maximum beam intensity of 0.75 mA, used as a proton driver to supply an ISOL system with a UCx Direct Target able to sustain a power of 10 kW and produce neutron-rich ions at intensities 1 order of magnitude higher than existing facilities. The second exit will be used for applied physics: radioisotope production for medicine and neutrons for materials study. Quasi-mono-energetic neutrons at energies ranging 30–70 MeV will be produced using a 10 mA proton beam at an expected intensity around 5·10^5^ n·cm^−2^·s^−1^ at 3 m from the Li production target. The layout of the facility is shown in Figure 8. The proton beam from the cyclotron can be sent to two ISOL target caves (ISOL1 and ISOL2), three caves for radioisotopes production (RIFAC) and developments (RILAB), and an area for neutron production and materials study. SPES was designed to pursue also the aim of studying the production of innovative radionuclides for medicine (LARAMED) starting from the assumption that new radioisotopes may show unprecedented biological properties. Nonstandard radionuclide production is a fundamental opportunity for nuclear medicine in order to identify new radiopharmaceutical classes for diagnostic and therapeutic applications. RILAB will be dedicated to research in the field of radioisotopes (cross-section measurements, high-power target tests, etc.), whereas RIFAC will be devoted to the production of novel radioisotopes (^64^Cu, ^67^Cu, ^82^Sr, ^68^Ge, etc.). In June 2018, the INFN board of directors has approved the contracts for the supply of beam and the lease of laboratory space to BEST Theratronics for the commercial production of radioisotopes, initially using the ISOL2 cave. Also, in the field of nuclear medicine, the ISOLPHARM project will exploit the ISOL technique to produce a large variety of carrier-free radioisotopes with high radionuclidic purity (INFN international patent). The layout of SPES was designed in such a way as to operate two targets at the same time, distributing the beam according to a schedule that minimizes the radiation problems. It should be considered that the activation of materials at a beam power of 20–30 kW does not allow operating the same target for a long time. Considering a shift of 2 weeks with 2 days for beam preparation, 12 days of beam on target, and seven shifts for maintenance, we can offer about 5,000 h/year of beam dedicated to the ISOL targets and 5,000 for applications.

INFN has also collaborated on the construction of the experimental vault for dedicated biomedical research in the National Center for Oncological Hadrontherapy (CNAO) in Pavia. CNAO [76, 77] is one of the four centers in Europe in which hadrontherapy is administered with both protons and carbon ions. The main accelerator is a 25-m-diameter synchrotron designed to accelerate ions injected at 7 MeV/u up to the maximum energy corresponding to the magnetic rigidity of 6.35 Tm. For C^6+^ ions, this corresponds to 400 MeV/u; in the case of protons, the maximum available energy of 250 MeV corresponds to a magnetic rigidity of 2.43 Tm, well below the technically achievable maximum. For other ions that will be produced with a dedicated third source presently under construction, the maximum rigidity would still be 6.35 Tm, and the corresponding particle range would be determined by their charge and mass.

**Figure 9:**
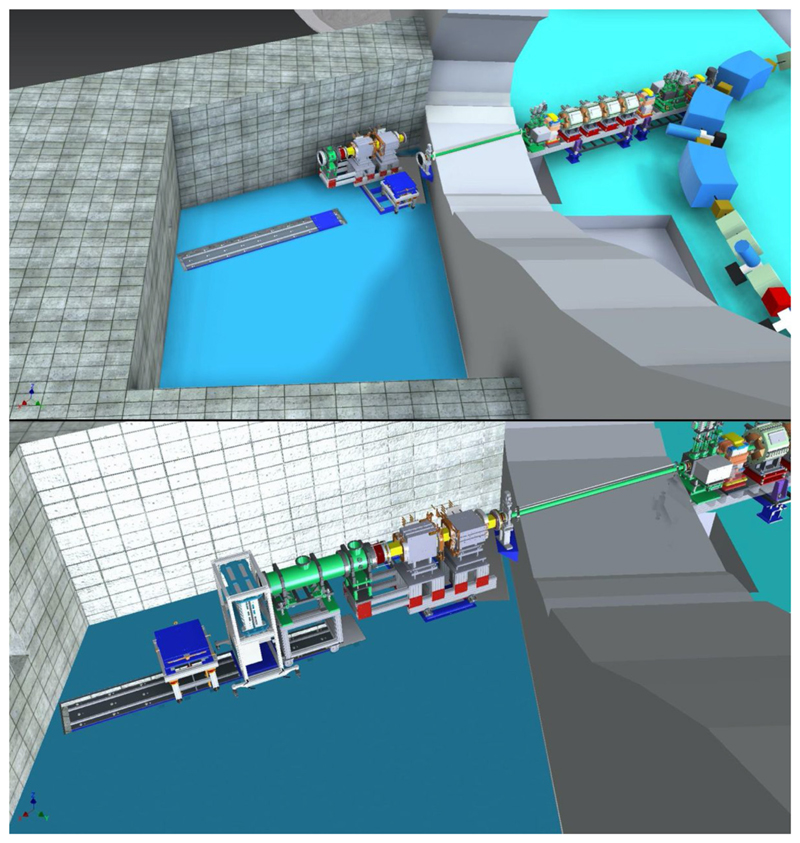
The experimental room at CNAO can be arranged in different configurations according to the experiment requirements.

CNAO has a 2-fold institutional purpose including both therapy and research, and it also provides great opportunities to perform various research activities related to radiation biophysics, radiobiology, space research, and detector development. For researchers, a dedicated experimental irradiation room is available in time slots not impacting patients’ treatment but specifically devoted to research purposes (i.e., some night shifts and weekends, typically) and, if applicable, in a parasitic modality during daily treatments, for the experiments in which the duration is not important and the measurement itself can be “paused” for an indefinite time. The beam distribution in the CNAO experimental room is based on the same active system in use in the treatment rooms. According to the needs of the experiment to be performed, the experimental beamline can be arranged in four different configurations depending on the space required downstream the target or the dimensions of the scanning field (Figure 9). The beam intensities available range from the clinical ones (<10^10^ protons per spill and < 4·10^8^ carbon ions per spill) down to a few particles per second.

CNAO offers the opportunity to external researchers to use its beams to perform basic and preclinical studies and to take advantage of a cell culture laboratory for sample preparation and processing. Thanks to a strong collaboration with the University of Pavia, in CNAO, it is also possible to carry out *in vivo* irradiations with small rodents, taking advantage of the nearby animal house facility, after technical evaluation and approval by the local ethical committee. Typical activities carried on at CNAO are development and test of beam monitors and of dosimeters, the development of the dose delivery system to improve the scanning technique (e.g., 4D treatments), the verification of dose delivered to the target, and of course radiobiology. The main topics for the present radiobiological research in CNAO comprise tissue, cell, and molecular experimental activities aiming to investigate the mechanisms of response after particle irradiation. In particular, one of the interests is modulation of the malignant behavior of surviving tumor cells by reducing or promoting their invasiveness or migration. Cellular and molecular mechanisms of radioresistance after irradiation with carbon ions, immune stimulatory effects of radiation, and immunosuppressive properties of high-LET radiations and abscopal effect are also subjects being studied at CNAO. One hot topic for CNAO radiobiological research is the evaluation of existing and/or new radiosensitizing agents with high-LET radiations. Physical amplification of LET by nuclear interaction, e.g., of protons on boron nuclei is also being studied.

This subject might become even more interesting since CNAO is willing to build a new boron neutron capture therapy (BNCT) facility in the next future. At CNAO, the introduction of an accelerator-based BNCT system is indeed presently under consideration. This activity will be performed, strengthening collaborations with INFN, University of Pavia, and other institutions, since the introduction of BNCT requires a properly structured multidisciplinary research phase with distributed skills (medical doctors, radiobiologists, medical physicists, chemists, etc.). Furthermore, the BNCT needs the development of biomedical imaging techniques for the mapping of the biodistribution of compounds enriched in ^10^B and for the selection of the ideal time interval of irradiation with thermal neutrons [78, 79].

## HIT

The Heidelberg Ion-Beam Therapy Center (HIT) at the Hospital of the University of Heidelberg (UKHD) is the first dedicated and hospital-based particle therapy center in Europe offering clinical scanned proton and carbon ion beams [80]. The treatment with helium ions is planned to start in late 2021, and oxygen beams are offered for preclinical research. Dose delivery is based on the intensity-controlled raster scanning method. The maximum field size is 200 × 200 mm^2^. The first worldwide rotating carbon ion gantry could be realized at HIT. Today, about 40% of patient treatments are executed at this unique device that combines robotic patient positioning, raster scanning dose delivery, and video-based patient tracking.

HIT has started routine patient treatment at a horizontally fixed beamline in November 2009. The carbon ion gantry is in clinical use since October 2012. In total, about 6,200 patients have been treated at HIT. It is an extension to the already available oncological methods at the Heidelberg University Hospital and indirectly complements the existing radiotherapy department hosting seven electron accelerators including tomotherapy and a gamma knife. In addition to patient care, a broad research program in the area of radiation oncology and accelerator physics, medical physics, and biophysics [81–83], annually using about 1,000 h of beamtime, is pursued at HIT. Large-scale clinical studies in the field of ion beam therapy as well as methodological studies are conducted here [84].

HIT operates an irradiation facility for preclinical research that delivers four ion species: protons and helium, carbon, and oxygen ions. HIT’s accelerator system provides energies up to 430 MeV/u for helium, carbon, and oxygen ions and up to 480 MeV for protons. For all ions, energy libraries are established that allow for millimeter-range steps within the therapeutic window (Bragg peak depth between 2 and 32 cm in water). For protons and helium ions, higher ranges/energies can be offered for research purposes. Within the center, laboratories for medical physics and experiment preparation as well as a dedicated rodent housing are located directly at the research cave. Laboratories for radiobiology are hosted in the attached building for conventional radiation therapy.

## KVI-CART

The core of the accelerator facility at the KVI-Center for Advanced Radiation Technology (KVI-CART), University of Groningen (UG), the Netherlands, is the superconducting cyclotron AGOR [85], built in collaboration with the Institut de Physique Nucléaire (Orsay, France) and operational in Groningen since 1996. It accelerates ion beams of all elements to a variable energy. Initially designed for research in nuclear physics and fundamental interactions, the focus of the research at the facility has, in relation with the establishment of a clinical proton therapy clinic at the University Medical Center Groningen (UMCG), in recent years shifted toward the radiation physics and biology of particle therapy.

Access to the facility is governed by the guidelines set out in the European Charter for Access to Research Infrastructures^14,15^. Since 1998, research on normal tissue damage in radiotherapy is performed in collaboration with the Radiation Oncology and Cell Biology departments of the UMCG. The experiments use mainly proton and carbon beams and have two main focal points: *in vivo* studies of non-local effects in (partial) irradiations of organs such as parotid [86], heart–lung system [87, 88], and neural tissues [89] and *in vitro* studies of various aspects of the radiation response of stem cells [90, 91].

**Figure 10:**
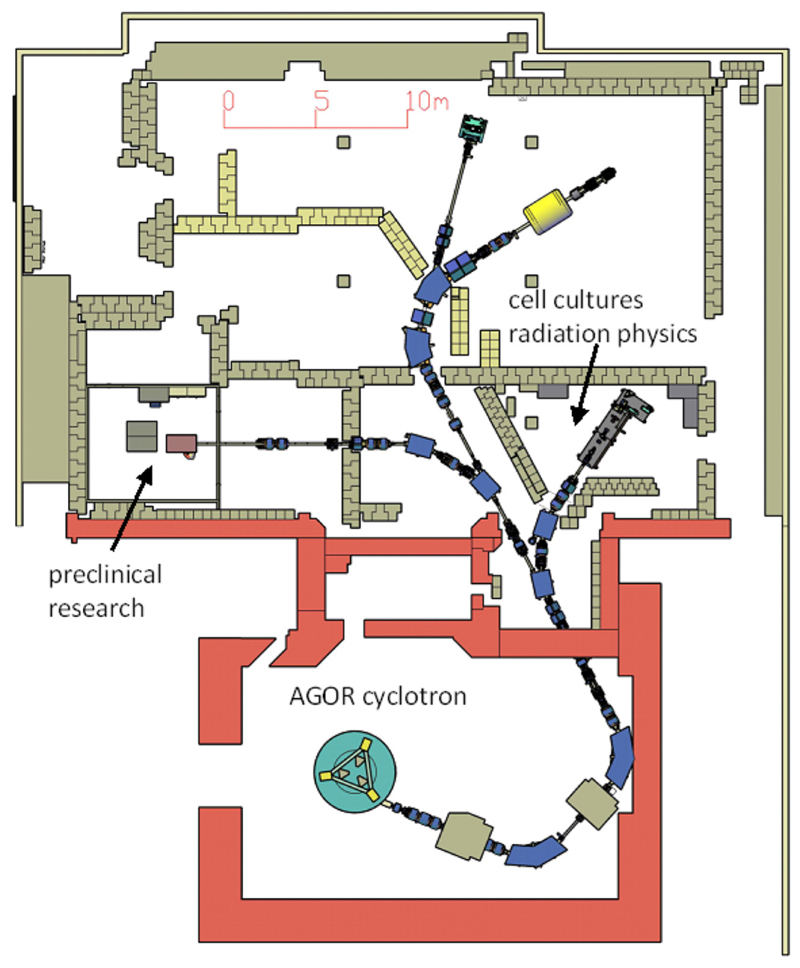
Floor plan of the AGOR accelerator facility with the new infrastructure for image-guided preclinical research.

For this research, a versatile, modular beamline [92] has been built that is also used for experiments in medical radiation physics and for radiation hardness testing with both protons and various heavy ions. The on-site laboratory facilities for the radiation biology research comprise an animal accommodation for wild-type rodents and two laboratories equipped with CO_2_ incubators and flow cabinets. In the coming years, the capabilities and capacity for radiation biology research will be substantially expanded. Currently, an additional beamline specifically for *in vivo* studies, equipped with 3D X-ray and bioluminescence imaging and 2D proton radiography at the irradiation position and funded by the Dutch cancer society KWF, is under development. In Figure 10, the floor plan of the accelerator facility, showing both the existing beamline for biomedical experiments and the new beamline, is displayed. With this new infrastructure, among others, more detailed studies of the spatial differentiation of the radiation response of normal and tumor tissues, interaction between particle irradiation, and systemic therapy, and biological effectiveness will be performed. At this new beamline, small animal irradiations will be performed with proton and helium beam as well as X-rays using different irradiation modalities. Besides the shoot-through method [89] employing 150–190 MeV protons, spread-out Bragg peak irradiations using primary beam energies up to 90 MeV/amu (range in water at 60 mm for both protons and helium) can also be performed. Both passive scattering and pencil beam scanning will be available, and the irradiations can be CW or pulsed with variable pulse duration (≥ 10 μs) and a frequency of up to 2 kHz. The design calculations for the beamline indicate that pencil beams with 0.5 mm FWHM are feasible. Based on preliminary experiments, local dose rates up to at least 1,000 Gy/s should be achievable for both proton and helium irradiations in pencil beam scanning.

In conjunction with this new infrastructure, additional animal accommodations with associated laboratories will be built to provide optimal research conditions. The capacity of the laboratories for *in vitro* research will also be expanded. A setup for live-cell confocal microscopy immediately after irradiation is under development in collaboration with Amsterdam UMC.

To facilitate the use of the new infrastructure by external users, we will, in collaboration with the central animal research facility of UG and UMCG, offer a “one stop shop” service. Based on the detailed experiment design developed in collaboration with the users, we will arrange the required Dutch authorizations, procure the required animals, perform the irradiations, and, when desired by the user, perform the post-irradiation followup experiments. The data will be provided to the users through a research data management platform controlled by the users.

The current radiation physics research by both internal and external users focuses on near-real-time *in vivo* range verification in particle therapy [93, 94] and various aspects of dosimetry, including characterization of the LET distribution of particle beams [95] and tissue relative stopping powers [96].

## Conclusions

Biomedical research programs at particle accelerators cover a vast range of topics such as particle therapy, radioisotope production for medicine, and radioprotection in space. Along with accelerator facilities with long tradition, there are several new accelerators now under construction that can enrich the nuclear physics weaponry for biological and medical research (Table 1). Collaboration is a key point to exploit the translational potential of these researches and maximize the benefit for patients. Only a strong network of different centers can exploit synergies, avoid duplications, and raise the quality and the impact of biomedical research at accelerators. To this aim, the successful model of the large high-energy physics experiment could be also adopted in the applied nuclear physics community. The International Biophysics Collaboration [37] has the ambition and the potentiality to provide such a network to foster collaborations, exchange of hardware, design of innovative research programs, and support for funding applications. Such a large collaboration will help research at accelerators to maintain its extraordinary role as a nuclear physics tool for biology and medicine.

## Figures and Tables

**Table 1:** A comparison of the accelerator facilities in the Biophysics Collaboration.

Name	Status	Location	Accelerator	Ions	Maximum energy
FAIR	Under construction (starts 2025)	Darmstadt, Germany	Synchrotron (100 Tm)	H to U	~10 GeV/n
GSI	In operation in FAIR-phase-0	Darmstadt, Germany	Synchrotron (18 Tm)	H to U	~1 GeV/n
NICA	Under construction	Dubna, Russia	Synchrotron	Up to Au	Up to 4.5 GeV/n for Au, up to 800 MeV/n for biomedical applications
iThemba	In operation; under upgrade	Cape Town, South Africa	Cyclotron	H	200 MeV. A 70 MeV cyclotron will be used for isotopes
HIMAC	In operation	Chiba, Japan	Synchrotron	He to Fe	~400 MeV/n for C-ions
GANIL	In operation	Caen, France	Cyclotrons	H to U	95 MeV/n
MI2B	In operation	France	Network of different small accelerators	H, He	~70 MeV
SPES	Under construction at LNL-INFN	Legnaro, Italy	Cyclotron	H	70 MeV
LNS-INFN	In operation; under upgrade	Catania, Italy	Cyclotron	H to Au	80 MeV/n (H to Ne), 50 MeV/n (Au)
CNAO	In operation	Pavia, Italy	Synchrotron	H and C	250 MeV (H), 400 MeV/n (C)
HIT	In operation	Heidelberg, Germany	Synchrotron	H, He, C, and O	480 MeV (H), 430 MeV (He to O)
KVI-CART	In operation	Groningen, The Netherlands	Cyclotron	H to Pb	190 MeV (H), 90 MeV/n (He to O), 75 MeV/n (Ne)

Highlighted in yellow are the facilities with clinical operation.

## Footnotes

1
www.gsi.de/bio-coll

2
www.gsi.de/IBER

3
www.gsi.de/BARB

4
https://www.ganil-spiral2.eu/scientists/ganil-spiral-2-facilities/available-beams/

5
https://www.mi2b.fr/

6
http://www.cenbg.in2p3.fr/-AIFIRA-Home-?lang=en

7
http://www.iphc.cnrs.fr/-Cyclotron-CYRCE-.html

8
http://www.iphc.cnrs.fr/-PRECy-.html

9
https://www.arronax-nantes.fr/

10
http://www.lnl.infn.it/index.php/en/accelerators-3/an-2000

11
http://www.lnl.infn.it/index.php/en/accelerators-3/cn

12
http://www.lnl.infn.it/index.php/en/accelerators-3/tandem-xtu

13
https://web.infn.it/spes/index.php/home/spe

14
https://ec.europa.eu/research/infrastructures/pdf/2016_charterforaccessto-ris.pdf#view=fit&pagemode=none

15
https://www.rug.nl/kvi-cart/research/facilities/agor/
